# Short leukocyte telomeres predict 25-year Alzheimer's disease incidence in non-*APOE* ε4-carriers

**DOI:** 10.1186/s13195-021-00871-y

**Published:** 2021-07-15

**Authors:** Fernanda Schäfer Hackenhaar, Maria Josefsson, Annelie Nordin Adolfsson, Mattias Landfors, Karolina Kauppi, Magnus Hultdin, Rolf Adolfsson, Sofie Degerman, Sara Pudas

**Affiliations:** 1 Department of Integrative Medical Biology, Umeå University, SE-901 87 Umeå, Sweden; 2 Umeå Center for Functional Brain Imaging, Umeå University, SE-90 187 Umeå, Sweden; 3 Department of Statistics, USBE, Umeå University, SE-901 87 Umeå, Sweden; 4 Center for Ageing and Demographic Research, Umeå University, SE-901 87 Umeå, Sweden; 5 Department of Clinical Sciences, Umeå University, SE-901 85 Umeå, Sweden; 6 Department of Medical Biosciences, Pathology, Umeå University, SE-901 85 Umeå, Sweden; 7 Department of Medical Epidemiology and Biostatistics, Karolinska Institute, SE-171 77 Stockholm, Sweden; 8 Department of Clinical Microbiology, Umeå University, SE-901 85 Umeå, Sweden

**Keywords:** Leukocyte telomere length, Dementia, Risk factors, Time-to-event analysis, Competing risks, Vascular dementia, Death

## Abstract

**Background:**

Leukocyte telomere length (LTL) has been shown to predict Alzheimer’s disease (AD), albeit inconsistently. Failing to account for the competing risks between AD, other dementia types, and mortality, can be an explanation for the inconsistent findings in previous time-to-event analyses. Furthermore, previous studies indicate that the association between LTL and AD is non-linear and may differ depending on apolipoprotein E (*APOE*) ε4 allele carriage, the strongest genetic AD predictor.

**Methods:**

We analyzed whether baseline LTL in interaction with *APOE* ε4 predicts AD, by following 1306 initially non-demented subjects for 25 years. Gender residualized LTL (rLTL) was categorized into tertiles of short, medium, and long rLTLs. Two complementary time-to-event models that account for competing risks were used; the Fine-Gray model to estimate the association between the rLTL tertiles and the cumulative incidence of AD, and the cause-specific hazard model to assess whether the cause-specific risk of AD differed between the rLTL groups. Vascular dementia and death were considered competing risk events. Models were adjusted for baseline lifestyle-related risk factors, gender, age, and non-proportional hazards.

**Results:**

After follow-up, 149 were diagnosed with AD, 96 were diagnosed with vascular dementia, 465 died without dementia, and 596 remained healthy. Baseline rLTL and other covariates were assessed on average 8 years before AD onset (range 1–24). *APOE* ε4-carriers had significantly increased incidence of AD, as well as increased cause-specific AD risk. A significant rLTL-*APOE* interaction indicated that short rLTL at baseline was significantly associated with an increased incidence of AD among non-*APOE* ε4-carriers (subdistribution hazard ratio = 3.24, CI 1.404–7.462, *P* = 0.005), as well as borderline associated with increased cause-specific risk of AD (cause-specific hazard ratio = 1.67, CI 0.947–2.964, *P* = 0.07). Among *APOE* ε4-carriers, short or long rLTLs were not significantly associated with AD incidence, nor with the cause-specific risk of AD.

**Conclusions:**

Our findings from two complementary competing risk time-to-event models indicate that short rLTL may be a valuable predictor of the AD incidence in non-*APOE* ε4-carriers, on average 8 years before AD onset. More generally, the findings highlight the importance of accounting for competing risks, as well as the *APOE* status of participants in AD biomarker research.

**Supplementary Information:**

The online version contains supplementary material available at 10.1186/s13195-021-00871-y.

## Introduction

Two thirds of dementia cases are diagnosed with Alzheimer’s disease (AD), characterized by neuronal deposition of amyloid-β plaques and neurofibrillary tau tangles, inflammatory activation of glia, reduced synaptic capacity, and neuronal loss [1]. These pathologic processes in the brain emerge from interactions among genetic and lifestyle factors [2]. AD has a long prodromal phase, as suggested by amyloid-β deposition that may start 15 years before the onset of dementia symptoms in some individuals [3]. Thus, successful prevention and treatment strategies require accurate prediction of individuals’ risk of the disease.

The apolipoprotein E (*APOE*) ε4 allele is the strongest genetic predictor of AD [1, 4], although among autopsy- or biopsy-confirmed AD cases the proportion of individuals not carrying the ε4 risk-allele ranges from 35 to 57% [5], which highlights the need for additional predictive markers. To date, only approximately 29% of AD heritability can be estimated by genome-wide association studies, whereas the *APOE* ε4 allele alone accounts for 24% [6]. Due to its well-documented relationship with cellular aging [7, 8], telomere length is a proposed biomarker of mortality and aging-related diseases such as dementia. Telomeres are protein-DNA complexes at the chromosome ends that prevent loss of coding DNA, as chromosomes are shortened with every cell division due to the “end replication problem” [8, 9]. Some cells, i.e., stem cells and germ cells escape telomere shortening by activating the telomerase enzyme complex, which adds telomeric repeats to the chromosome ends [8, 9]. However, telomeres may also shorten as a consequence of oxidative stress and inflammation processes derived from lifestyle factors [8, 9]. There is robust evidence from large-scale studies and meta-analyses associating leukocyte telomere length (LTL) shortening with aging, aging-related diseases, and mortality [10–13]. Even though short LTL is predictive of these events and processes, it is yet to be established whether it is a cause, consequence, or mere correlate of them [14].

LTL’s association with dementia, and more specifically with AD risk, is inconsistent. Case-control and meta-analytic evidence based on case-control studies demonstrate short LTL in individuals diagnosed with AD [15–19], while other similar studies found no associations [20–23]. Furthermore, cross-sectional case-control study designs cannot estimate the potential role of LTL as a predictive AD risk marker if pre-diagnosis measurements are not available. Reports on LTL association with AD using prospective time-to-event analyses also show conflicting results. Short baseline LTL has been associated with a higher probability to develop AD [24] and all-cause dementia [10], while null associations with AD have also been found [25]. Noteworthy, another longitudinal time-to-event analysis study found a non-linear LTL association with AD, with both short and long LTL being associated with elevated AD risk [26]. In accordance, a similar short and long LTL risk association was observed for amnestic mild cognitive impairment, considered a prodromal stage to AD [27]. Such non-linearity may have led to divergent results or may have precluded observing significant associations between LTL and AD in the past. Other limitations of prior time-to-event studies, such as limited sample sizes, ranging from 20 to 81 demented participants [24, 25, 28], limited follow-up times, ranging from 2.5 to 11 years [10, 24–26, 28], or not accounting for genetic and lifestyle factors [24] may also underlie divergent findings.

Another reason behind discrepant results could be that the abovementioned studies employing time-to-event analyses to estimate associations between LTL and AD have not accounted for competing risks. However, the well-established association between short LTL and the risk of death [12] is a clear competing risk, especially in studies with a long follow-up time. Consequently, participants with short LTL will be removed from the AD risk set because of death, which may impede the detection of significant associations between LTL and AD. Accordingly, when subjects are diagnosed with another dementia disorder they are also removed from the AD risk set. For this reason, competing risk time-to-event models may access LTL association with AD not detected in classical time-to-event analysis.

Group-level LTL-AD associations may also be obscured by heterogeneous associations for certain subgroups. Previous studies on LTL associations with AD [26] and age-related cognitive decline, a possible prodromal symptom of dementia [29–32], have observed interactions between LTL and *APOE* ε4, such that LTL is more strongly associated with AD in *APOE* ε4-carriers. This suggests that LTL may predict AD and cognitive decline differently among carriers and non-carriers of *APOE* ε4.

The present study aims to investigate whether baseline LTL, alone or in interaction with *APOE* ε4, predicts the onset of AD in a well-characterized population-based sample of older individuals followed for 25 years [33, 34]. To achieve this, we performed time-to-event analyses controlling for lifestyle-related markers of obesity, diabetes, hypertension, and inflammation, as lifestyle factors affect both dementia progression [33, 35, 36] and TL dynamics [7, 8]. We employed two complementary time-to-event models accounting for competing risks of mortality and vascular dementia (VaD), as opposed to the standard Cox regression model [37–39]. First, the Fine-Gray model [40, 41] was used to assess the effect of LTL on AD incidence, which reflects covariate effects on the expected proportion of subjects with AD in the population over time. Second, the cause-specific hazard model [37] was used to estimate the effect of LTL on the specific risk of AD and reflects covariate effects on the instantaneous rate of occurrence in individuals who are currently alive and dementia-free. By considering both models side-by-side, we obtain a more complete understanding of the effect of LTL on competing risk endpoints. Notably, to our knowledge, the present study is the first one accounting for competing risks to evaluate LTL for AD prediction.

## Methods

### Study population

The Betula project is a longitudinal population-based prospective study initiated in 1988 (total *n* = 4425), with the objectives to examine cognition, health, social, and physiological parameters from adulthood to older age [33, 34]. The recruitment procedures have been extensively described elsewhere [34, 42], but participants were required to be non-demented native Swedish speakers without congenital or acquired intellectual disabilities, or severe hearing/vision impairments at recruitment. The observation scheme in the study is fixed, in which individuals are examined at five years intervals (T1–T7 test waves). The presence of dementia has been evaluated adjacent to each test wave, most recently in 2016/2017.

### Clinical characterization and dementia diagnosis assessments

Dementia diagnoses were based on multiple sources of clinical information, comprising written, and computerized medical records, supplemented by outcomes from the Betula study health and cognitive assessments (for detailed description see [33]). The Diagnostic and Statistical Manual of Mental Disorders 4th edition (DSM-IV) was used for dementia classification [43].

All diagnosed AD and VaD cases showed a progressive cognitive and functional decline as evident by symptoms attributable to each dementia type. Participants receiving an AD diagnosis showed an insidious onset and progressive cognitive decline as well as other symptoms typically attributable to clinical AD. Individuals with cardiovascular burden accompanied with neurological signs and a fluctuating cognitive symptomatology with stepwise progression were diagnosed with VaD. Less common dementia disorders such as Parkinson’s disease, Lewy body dementia, frontotemporal dementia, progressive supranuclear paralysis, and corticobasal degeneration were always extensively examined by the Departments of Geriatric Medicine and Neurology, and diagnoses were set using established criteria [44]. Individuals presenting symptoms of cognitive impairment close to death, often accompanied by severe somatic conditions and delirious episodes, were not diagnosed as demented; neither were individuals exhibiting a long-term low cognitive capacity after e.g., trauma, tumor, or subarachnoid hemorrhage.

### Inclusion and exclusion criteria

Participants aged 45 years or older (*n* = 1842) from samples 1 and 3 of the Betula project, enrolled at the second (1993–1995) test wave, were initially considered for the present study. The first test wave was not included here, as LTL was measured from the second test wave onwards. As the study intended to follow late-onset AD onset for participants not demented at study entry, participants with dementia diagnostic before the second test wave or participants with early onset of dementia (demented before being 60 years old, *n* = 1) were excluded, as well as individuals deceased at the year of study entry or at a young age (deceased before 60 years old, *n* = 21).

Subjects younger than 45 years old at baseline were excluded, as they were unlikely to develop dementia during the studied period. Other dementia types were also excluded due to low numbers precluding treating them as competing events, e.g., dementia not-otherwise specified (*n* = 15), dementia due to Parkinson’s disease (*n* = 5), Lewy body dementia (*n* = 6), frontotemporal dementia (*n* = 2), progressive supranuclear paralysis (*n* = 1), and corticobasal degeneration (*n* = 1). Lost to follow-up individuals (*n* = 121) were excluded, and comprised those that moved from the region, had insufficient assessment basis, or did not leave consent for reading their medical record. Subjects with missing values for telomere length (*n* = 107) and *APOE* genotyping (*n* = 45) were excluded from the final sample. *APOE* ε2/*APOE* ε4 genotype participants were not included in our study (*n* = 42, of which *n* = 6 were diagnosed with AD, and *n* = 3 with VaD). The reason for this was that in contrast to *APOE* ε4, the *APOE* ε2 allele may have a protective role in AD [45]. At the end of the selection procedure (see also Supplementary Fig 1), the final sample included 1306 individuals (see Table 1 for further description).

**Table 1:** Baseline characteristics and health markers among study groups (*n* = 1306)

	Healthy	Alzheimer's disease	Vascular dementia	Deceased
Number	596 (45.6%)	149 (11.4%)	96 (7.3%)	465 (35.7%)
Gender, male	249 (41.8%)	34 (22.8%)	42 (43.7%)	246 (37.1%)
Age at the event, years	76 (10) *	82 (9)	83 (7)	83 (13)
Age at study entry, years	55 (11)	71 (11)	71 (7)	75 (15)
rLTL	0.026 (0.16)	–0.045 (0.17)	–0.046 (0.18)	–0.055 (0.14)
*APOE* ε4-carriers	152 (25.5%)	78 (52.3%)	31 (32.3%)	102 (15.4%)
Serum cholesterol, mg/dL	250.96 (57.9)	270.27 (54.1)	262.55 (54.1)	254.83 (65.6)
Pulse pressure, mmHg	50 (20)	65 (25)	70 (20)	65 (25)
Plasma glucose, mg/dL	93.69 (16.2)	93.69 (14.4)	97.29 (21.6)	97.29 (18.1)
Sedimentation rate, mm/h	9 (8)	14 (15)	12.5 (11)	14 (12)
Lymphocyte proportion	0.316 (0.09)	0.306 (0.09)	0.277 (0.10)	0.290 (0.10)

Data are expressed as counts (percentage) or medians (interquartile range). *APOE* ε4 apolipoprotein E ε4, *rLTL* residualized leukocyte telomere length. *Age at the last-follow-up of event-free participants

### Leukocyte telomere length

Genomic DNA from peripheral blood leukocytes was used to measure the LTL, applying the Cawthon quantitative polymerase chain reaction (PCR) method with minor modifications [46, 47]. Briefly, separate telomere (TEL) and hemoglobin subunit beta (HBB) gene were used to calculate the T/S (TEL/HBB) values using the 2−ΔCt method, in which ΔCt = CtTEL-CtHBB. The relative LTL values were obtained by dividing the T/S value of each sample with the T/S value of DNA from the CCRF-CEM cell line as reference. A comprehensive protocol of normalizations and quality controls was employed, as described in detail in ref. [48]. All LTLs were measured in 2014. LTL from 626 samples of the third Betula test wave (1998–2000) were used to replace non-measured LTL in the second test wave.

### APOE genotyping and other covariates

*APOE* genotypes were determined by PCR (for a detailed description, see [49]). Resting diastolic and systolic pressure were assessed concomitantly with clinical lab tests for serum cholesterol, plasma glucose, erythrocyte sedimentation rate, and differential white blood cell counts. High serum cholesterol was considered when serum levels were ≥ 240 mg/dL [50]. Pulse pressure was calculated by subtracting diastolic pressure from systolic pressure. Blood lymphocyte proportion was calculated as lymphocyte count divided by the sum of all white blood cells count (sum of neutrophils, eosinophils, basophils, lymphocytes, and monocytes). All covariates were recorded from the baseline time-point.

### Statistical analyses

LTL was residualized against gender using a linear regression model, to remove variance associated with gender; hereafter referred to as residualized leukocyte telomere length (rLTL). Initial analyses revealed a nonlinear relationship between rLTL and AD risk, where the most parsimonious description of the rLTL profile was found for a tertile division, evidenced by natural splines (based on lowest Bayesian information criteria - BIC, see Supplementary Fig 2) [51]. For this reason, rLTL was used in the regression analyses divided in tertiles of length, where medium rLTL was used as the reference group for the short and long rLTL groups.

We employed two different time-to-event models, both accounting for competing risks. First, the *Fine-Gray model*, which estimates the subdistribution hazard function (and corresponding subdistribution hazard ratios) can be used to correctly predict the cumulative incidence function for an event. In the model, those who experienced a competing event are still in the risk set, and only those who experienced the event of interest or those who are truly censored (i.e., event-free at last follow-up) are removed [37, 40, 41]. The second model was *the cause-specific hazard model.* In contrast to the Fine-Gray model, the cause-specific hazard model estimates the instantaneous risk of an event among those subjects who are currently event-free and can be used to correctly assess the effect of covariates on the risk of an event. Here, those who have already experienced the event or who have experienced a competing event are no longer in the risk set [37, 38]. The equations for the subdistribution hazard and cause-specific hazard functions [37, 41], and the cumulative incidence function [41, 52] can be seen in the Supplementary material.

Time from baseline (in years) was used as the time scale. The time-to-event models were adjusted for lifestyle-related risk factors at baseline; high cholesterol, pulse pressure, plasma glucose, erythrocyte sedimentation rate, and lymphocyte proportion, while controlling for gender, age, and age squared. Carriers of the *APOE* ε4 allele, high cholesterol, and gender were included in the models as binary indicator variables. We restricted the number of selected independent variables to ten events-per-variable (EPV) ratio, combined with a backward selection of variables by the lowest Akaike information criteria (AIC) in the cause-specific hazard model for AD. To analyze if the effect of rLTL depends on the *APOE* ε4 allele carriage (considering both *APOE* ε3/ε4 and *APOE* ε4/ε4 genotypes as *APOE* ε4-carriers), we included interaction terms, which were included in the models when significant (*P* < 0.05). In addition, the proportional hazards assumption was assessed by testing for time-by-covariate interactions in the multivariable analyses. Validation of the models used the area under the receiver operating characteristic (ROC) curve (AUC) over the study time-course, assessing the prediction ability of both Fine-Gray and cause-specific models. Bootstrap cross-validation was based on 100 bootstrap samples. An AUC above 0.8 indicates a model with good discriminatory accuracy [53]. Competing risk analyses, validation, and plots were carried out using the *cmprsk*, *ggplot2*, *riskRegression*, *splines*, and *survival* packages in R (RStudio Inc. Vesion 1.2.5033, 2019) [52, 53].

## Results

After 25 years of follow-up, 596 individuals remained healthy, 149 were diagnosed with AD, 96 were diagnosed with VaD, and 465 non-demented individuals were deceased. The median age at baseline was 65 years (range 45–86 years), whereas the median age of AD onset was circa 82 years. This was similar for the competing risk events, as the median age at the VaD onset and at the time of death was 83 years (Table 1). The proportion of *APOE* ε4-carriers in the whole sample was 27.8% and, as expected, there was a higher prevalence of females (77.2%) and *APOE* ε4-carriers (52.3%) among AD cases (Table 1). The number of *APOE* ε4-carriers was similar among short (*n* = 127), medium (*n* = 100), and long (*n* = 136) rLTL groups (Supplementary Table 1).

### LTL effect on AD incidence

Fine-Gray models were first used to estimate the incidence of AD while considering VaD and death as competing events. Model validation using AUC curves over the study time-course showed that the full model had good discriminatory accuracy (> 0.8; Supplementary Fig 3). In the model, *APOE* ε4 and short rLTL were significantly associated with an increased incidence of AD (Table 2). In addition, significant interactions were present for short and long rLTL and *APOE* ε4, as well as for short rLTL and time. The significant covariate-covariate interactions between both short and long rLTL with *APOE* ε4 evidence that rLTL predicts AD incidence differently among *APOE* ε4-carriers and non-carriers (Table 2). For a clearer interpretation of the rLTL-*APOE* interaction, we repeated the Fine-Gray model dummy-coding short, medium, and long rLTL groups among *APOE* ε4-carriers and non-carriers into six separate groups (see Supplementary Table 2). With medium rLTL as reference group, both short and long rLTL showed an increased AD incidence for non-*APOE* ε4-carriers, although only statistically significant for short rLTL (Table 2 and Supplementary Table 2). Among the carriers of the *APOE* ε4 allele, these associations were inverted, as both short and long rLTL had a tendency of association with a decreased AD incidence when compared with medium rLTL *APOE* ε4-carriers (see Supplementary Table 2).

**Table 2:** Fine-Gray models predicting the incidence of AD, VaD, and death [sHR (95% CI); *P* value] (*n* = 1306)

	Alzheimer’s disease (*n* = 149)	Vascular dementia (*n* = 96)	Death (*n* = 465)
Short rLTL	3.24 (1.404–7.462); *P* = 0.005	1.09 (0.668–1.780); *P* = 0.72	1.82 (1.234–2.682); *P* = 0.002
Long rLTL	1.42 (0.751–2.697); *P* = 0.28	1.27 (0.758–2.120); *P* = 0.37	0.82 (0.637–1.056); *P* = 0.12
*APOE* ε4-carriers	6.61 (3.592–12.168); *P* < 0.0001	1.28 (0.841–1.960); *P* = 0.26	0.70 (0.556–0.882); *P* = 0.002
Short rLTL/*APOE* ε4 interaction	0.41 (0.181–0.920); *P* = 0.03	–	–
Long rLTL/*APOE* ε4 interaction	0.40 (0.169–0.948); *P* = 0.03	–	–
High cholesterol (≥ 240 mg/dL)	1.62 (1.061–2.463); *P* = 0.02	1.15 (0.736–1.830); *P* = 0.55	1.82 (1.234–2.682); *P* = 0.002
Pulse pressure, mmHg	0.99 (0.986–1.006); *P* = 0.47	1.02 (1.005–1.030); *P* = 0.005	0.82 (0.637–1.056); *P* = 0.12
Plasma glucose, mg/dL	0.99 (0.980–1.000); *P* = 0.04	1.00 (0.994–1.010); *P* = 0.97	0.70 (0.556–0.882); *P* = 0.002
Sedimentation rate, mm/h	0.99 (0.984–1.014); *P* = 0.89	1.03 (0.977–1.040); *P* = 0.03	1.03 (1.017–1.042); *P* < 0.0001
Lymphocyte proportion	0.002 (0.00004–0.156); *P* = 0.004	0.19 (0.012–2.870); *P* = 0.25	0.58 (0.166–2.057); *P* = 0.40
Gender, male	0.37 (0.249–0.552); *P* < 0.0001	2.35 (0.666–3.600); *P* = 0.03	1.87 (1.531–2.274); *P* < 0.0001
Age at baseline, years	2.29 (1.719–3.051); *P* < 0.0001	3.64 (2.571–23.640); *P* < 0.0001	1.21 (1.087–1.345); *P* = 0.0004
Age squared	0.995 (0.992–0.997); *P* < 0.0001	0.991 (0.981–0.990); *P* < 0.0001	0.999 (0.998–1.000); *P* = 0.04
Short rLTL time interaction	0.92 (0.869–0.972); *P* = 0.003	–	0.95 (0.922–0.983); *P* = 0.002
Sedimentation rate time interaction	–	0.996 (0.995–1.000); *P* = 0.006	0.998 (0.997–0.999); *P* = 0.005
Lymphocyte proportion time interaction	1.76 (1.266–2.447); *P* = 0.0007	–	–
Gender time interaction	–	0.92 (0.850–0.986); *P* = 0.02	–
	Pseudo likelihood ratio = 241	Pseudo likelihood ratio = 123	Pseudo likelihood ratio = 480

*APOE ε4* apolipoprotein E ε4, *CI* confidence interval, *rLTL* residualized leukocyte telomere length, *sHR* ratio of the subdistribution hazards of Fine-Gray model, accounting for competing risks. Time from baseline, in years, was used as the time scale

**Fig. 1:**
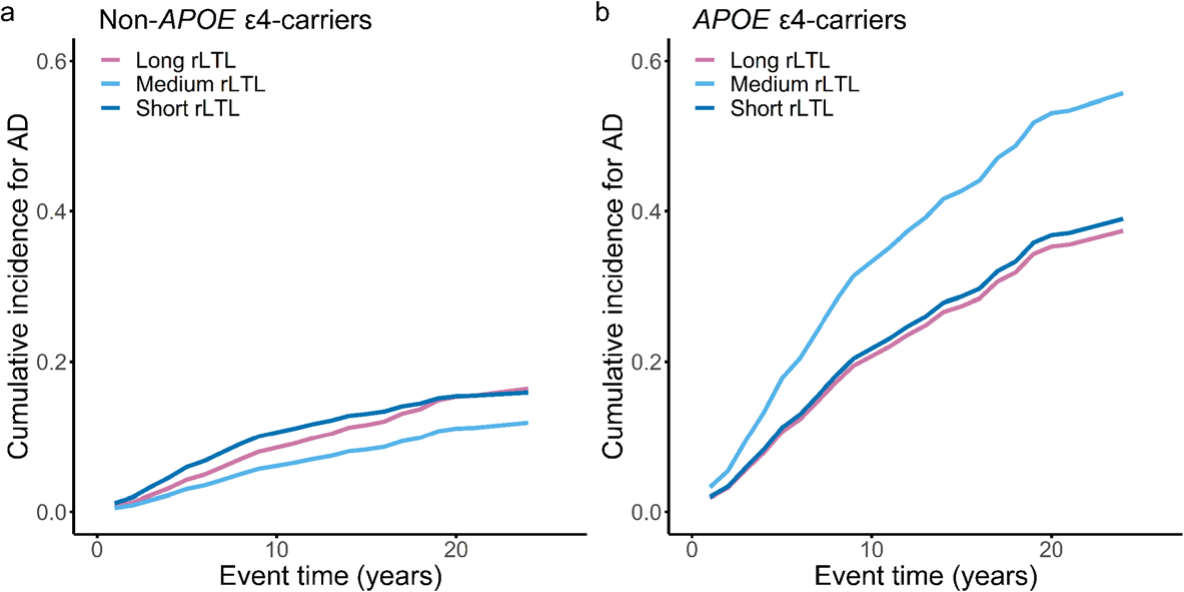
Cumulative incidence plots estimating the incidence of individuals progressing to Alzheimer’s disease (AD) for (**a)** non-apolipoprotein E ε4-carriers (non-*APOE* ε4-carriers) and (**b**) *APOE* ε4-carriers according to residualized leukocyte telomere length (rLTL) tertiles. The Fine-Gray hazard function was estimated for a representative 65-year-old female with high levels of cholesterol (> 240 mg/dL), and median values of pulse pressure, plasma glucose, erythrocyte sedimentation rate, lymphocyte proportion, and age squared, including time interactions for short telomere length and lymphocyte proportion in non-*APOE* ε4-carriers

This profile can be observed in the cumulative incidence plots from the Fine-Gray hazard function (Fig. 1). For a representative 65-year-old female non-*APOE* ε4-carrier, short and long rLTL increased the AD cumulative incidences when compared with medium rLTL (Fig. 1a); however, if she was a carrier of the *APOE* ε4 allele, the associations were inverted, and short and long rLTLs decreased AD cumulative incidences in comparison with the medium rLTL group (Fig. 1b; see also Supplementary Table 2). Moreover, we found a significant time-by-covariate interaction for short rLTL among non-carriers. This indicates that the estimated association of short rLTL with increased AD incidence is present at baseline, i.e., at time 0 (sHR = 3.24, CI = 1.404–7.462, *P* = 0.005), and decreases with time (sHR = 0.92, CI = 0.869–0.972, *P* = 0.003), because the time-interaction sHR < 1 (Table 2). As can be seen in the cumulative incidence plot, the elevated cumulative incidence close to baseline (i.e., ca. 0–5 years) attenuates over the study period for non-carriers with short rLTL (Fig. 1a). Finally, AUC curves were estimated to assess the prediction ability for models with and without rLTL for the non-*APOE* ε4-carriers (*n* = 943), which showed a small but consistent increase in prediction ability across the study (Supplementary Fig 4).

### LTL effect on the incidence of competing events

In the Fine-Gray model, short rLTL was also significantly associated with an increased incidence of death, but not with VaD. Notably, similar to the profile for AD, both short and long rLTL were non-significantly associated with increased VaD incidence in the Fine-Gray models for (sHR > 1). In contrast to these non-linear U-shaped associations for AD and VaD, short rLTL was significantly associated with an increased incidence of death, while long rLTL showed a non-significant trend of a decreased death incidence (Table 2), i.e., a linear association. Notably, there were no significant interactions between rLTL and *APOE* in the Fine-Gray models for VaD or death (Table 2).

### LTL effect on the cause-specific risk of AD

Subsequently, the cause-specific hazard model was used to assess the effect of covariates on the cause-specific risk of AD. Again, model validation using the AUC curve showed good discriminatory accuracy (> 0.8) across the study time-course (Supplementary Fig 3). Similar to the findings from the Fine-Gray model, *APOE* ε4-carriers presented a 6.64 times higher cause-specific risk of AD compared to non-carriers (Table 3). Short rLTL showed a non-significant trend of increased AD risk in non-carriers of *APOE* ε4 and, according to the model estimates, short rLTL increases the cause-specific risk of AD by 67% (Table 3). Significant interactions were also present for short and long rLTL and *APOE* ε4 in the cause-specific hazard model. The significant covariate-covariate interactions between both short and long rLTL with *APOE* ε4 evidence that rLTL predicts AD cause-specific risk differently among *APOE* ε4-carriers and non-carriers (Table 3). For a clearer interpretation of the rLTL-*APOE* interaction, we repeated the cause-specific hazard model dummy-coding the short, medium, and long rLTL groups among *APOE* ε4-carriers and non-carriers into six groups (Supplementary Table 2). In agreement with our Fine-Gray model findings, short and long rLTL showed a trend of a decreased cause-specific risk of AD among *APOE* ε4-carriers, when compared with medium rLTL *APOE* ε4-carriers (Supplementary Table 2).

**Table 3:** Cause-specific hazard models predicting the risk of AD, VaD, and death [csHR (95% CI); *P* value] (*n* = 1306)

	Alzheimer’s disease (*n* = 149)	Vascular dementia (*n* = 96)	Death (*n* = 465)
Short rLTL	1.67 (0.947–2.964); *P* = 0.07	1.29 (0.784–2.119); *P* = 0.31	1.17 (0.976–1.406); *P* = 0.08
Long rLTL	1.38 (0.728–2.615); *P* = 0.32	1.12 (0.657–1.912); *P* = 0.67	0.85 (0.688–1.047); *P* = 0.12
*APOE* ε4-carriers	6.64 (3.651–12.087); *P* < 0.0001	1.54 (0.992–2.379); *P* = 0.05	1.32 (1.115–1.575); *P* = 0.001
Short rLTL/*APOE* ε4 interaction	0.43 (0.197–0.945); *P* = 0.03	–	–
Long rLTL/*APOE* ε4 interaction	0.42 (0.178–0.984); *P* = 0.04	–	–
High cholesterol (≥ 240 mg/dL)	1.49 (1.001–2.243); *P* = 0.05	1.15 (0.730–1.827); *P* = 0.53	1.06 (0.902–1.261); *P* = 0.45
Pulse pressure, mmHg	1.00 (0.990–1.011); *P* = 0.89	1.02 (1.007–1.029); *P* = 0.001	1.005 (1.001–1.010); *P* = 0.01
Plasma glucose, mg/dL	0.99 (0.984–1.001); *P* = 0.08	1.002 (0.997–1.008); *P* = 0.38	1.003 (1.002–1.005); *P* = 0.0002
Sedimentation rate, mm/h	1.01 (0.992–1.023); *P* = 0.35	1.005 (0.985–1.026); *P* = 0.63	1.01 (1.008–1.022); *P* < 0.0001
Lymphocyte proportion	0.41 (0.042–4.104); *P* = 0.45	0.07 (0.004–1.097); *P* = 0.05	0.24 (0.083–0.672); *P* = 0.006
Gender, male	0.46 (0.306–0.691); *P* = 0.0001	1.16 (0.752–1.800); *P* = 0.49	0.85 (0.535–1.357); *P* = 0.50
Age at baseline, years	1.95 (1.448–2.633); *P* < 0.0001	3.36 (1.999–5.639); *P* < 0.0001	2.96 (1.807–4.841); *P* = 0.02
Age squared	0.991 (0.994–0.998); *P* = 0.0002	0.99 (0.989–0.996); *P* < 0.0001	0.993 (0.990–0.997); *P* < 0.0001
Gender time interaction	–	–	1.26 (1.028–1.555); *P* = 0.001
Age at baseline time interaction	–	–	0.73 (0.606–0.885); *P* < 0.0001
Age squared time interaction	–	–	1.002 (1.001–1.003); *P* = 0.001
	AIC = 1770	AIC = 1153	AIC = 7964

*AIC* Akaike information criteria; *APOE ε4* apolipoprotein E ε4; *CI* confidence interval; *csHR* cause-specific hazard ratio of cause-specific hazard model, accounting for competing risks; *rLTL* residualized leukocyte telomere length. Time from baseline, in years, was used as the time scale

**Fig. 2:**
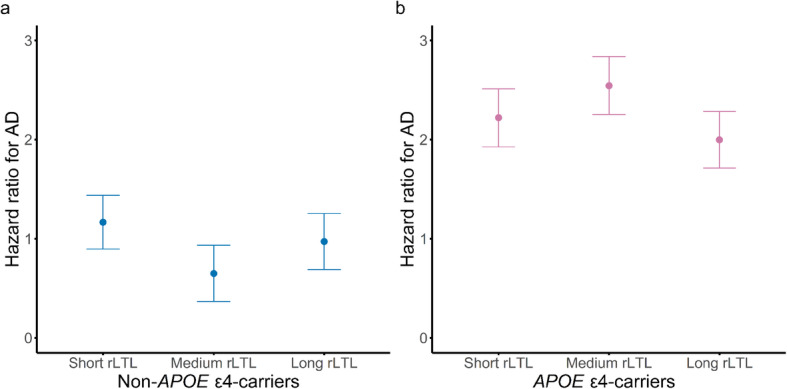
Cause-specific hazard ratio plot estimating the risk of individuals to progress to Alzheimer’s disease (AD) in (**a**) non-apolipoprotein E ε4-carriers (non-*APOE* ε4-carriers) and (**b**) *APOE* ε4-carriers, according to residualized leukocyte telomere length (rLTL) tertiles. The cause-specific hazard function was estimated for a representative female of 65 years old, with high cholesterol levels (> 240 mg/dL), and median values of pulse pressure, plasma glucose, erythrocyte sedimentation rate, lymphocyte proportion, and age squared

The cause-specific hazard ratios for AD from the model were plotted to better understand the associations of short and long rLTLs and AD risk, as well the interaction between rLTL tertiles and *APOE* ε4 allele. For a representative 65-year old female non-*APOE* ε4-carrier, the risk of AD is non-significantly higher if she has short and long rLTL compared with medium rLTL, with short rLTL showing a more pronounced risk of AD, in accordance with the trends from the cause-specific hazard model (Fig. 2 and Supplementary Table 2). However, if she was an *APOE* ε4-carrier, short and long rLTL shows a less pronounced risk of AD, being slightly but non-significantly lower when compared with medium rLTL (Fig. 2 and Supplementary Table 2). Supplementary Fig 4 shows the AUC curves for cause-specific models with and without rLTL for the *APOE* ε4 non-carriers, indicating a small but consistent improvement in prediction ability when including rLTL.

### LTL effect on the cause-specific risk of competing events

Cause-specific hazard models were also used to assess the effect of covariates on the risk of the competing risks events VaD or death (Table 3). In agreement with our Fine-Gray model findings (Table 2) and similar to AD, short and long rLTL were associated with numerically, but non-significantly, increased cause-specific risk of VaD (Table 3). In the association between rLTL and death, short rLTL had a non-significant trend of increased cause-specific risk and, accordingly, long rLTL had a trend of decreased death risk. According to the model estimates, short rLTL increased the cause-specific risk of death by 17% (Table 3).

### Covariate effects on the incidence and cause-specific risk of AD and competing events

Some covariates used to adjust the Fine-Gray and cause-specific hazard models showed significant associations with AD, VaD, and death. Although we did not have hypotheses regarding specific covariates, we report them for completeness. Statistically significant covariate associations with AD incidence were seen for high blood cholesterols, low plasma glucose, low lymphocyte proportion, female gender, and older age (Table 2). Similar covariate associations for female gender and higher age were found for the cause-specific risk of AD (Table 3). Some covariates also had significant associations with the incidence of VaD, such as high pulse pressure, high sedimentation rate, male gender, and older age (Table 2). Of these, high pulse pressure and older age were significant for the cause-specific risk of VaD (Table 3). Most of the analyzed covariates were significantly associated with death incidence, such as high cholesterol, low plasma glucose, high sedimentation rate, male gender, and older age (Table 2). *APOE* ε4 carriage was associated with a decreased incidence of death. For the cause-specific risk of death, significant covariate associations were found for high pulse pressure, high plasma glucose, high sedimentation rate, low lymphocyte proportion, and older age (Table 3). *APOE* ε4 carriage was associated with a higher cause-specific risk of death.

### Sensitivity and control analyses

To test potential bias from *APOE* ε2 putative protective role, we repeated the AD analyses excluding *APOE* ε2 homo- and heterozygotes (ε2/ε2 *n* = 8, ε2/ε3 *n* = 166), but no significant changes were found in the sHR and csHR profiles. Both competing risk time-to-event models were also repeated combining both dementia types to an all-cause dementia category (Supplementary Table 3), which rendered no significant associations between dementia and rLTL. The number of *APOE* ε4/ε4 homozygotes among short, medium, and long rLTL were 9, 8, and 9, respectively, evidencing that these participants with elevated AD risk were not overrepresented in any rLTL group.

Previous Betula project studies found longer LTL among *APOE* ε4-carriers [31, 54]. Here, after gender esidualization, the mean rLTL did not differ between *APOE* ε4-carriers vs. non-carriers (means of −0.009 and −0.004, respectively; *P* = 0.54).

## Discussion

After 25 years of follow-up of 1306 healthy participants older than 45 years, our findings indicate that short LTL significantly predicts an increased AD incidence in non-carriers of the *APOE* ε4 risk-allele, while also non-significantly increasing its cause-specific risk. A different hazards profile was seen among *APOE* ε4-carriers, in which both short and long LTLs showed a trend of a decreased incidence and cause-specific risk of AD. For our competing risk events, no evidence was obtained for an LTL association with VaD, while short LTL was significantly associated with an increased incidence of death, and borderline associated with an increased cause-specific risk of death.

Our finding on LTL as an AD predictor is in agreement with some previous studies [10, 24–26], but further elaborated on the nature of its predictive ability by showing that it was differentially predictive in non-carriers vs. carriers of the *APOE* ε4 allele. Past time-to-event studies observing divergent AD-LTL relationships may have been limited by not accounting for competing risks or non-linearity of risk-associations, limited sample sizes, and follow-up times, or failing to account for *APOE* interactions [10, 24–26, 28]. As in our analyses, *APOE* ε4 has consistently been found to be the strongest genetic predictor of AD [6]; however, the non-*APOE* ε4-carriers, which are more prevalent worldwide (69–94%) and constitute a sizeable proportion (35–57%) of confirmed AD-cases [5], remain without good predictive markers. Our findings thus hold clinical value in that they indicate that LTL may improve AD prediction for non-carriers. Thanks to our long follow-up time, we were able to measure LTL on average 8 years before AD onset (median: 8; min-max: 1–24 years), which further highlights the predictive value of LTL in relation to AD in non-*APOE* ε4-carriers. Moreover, our estimates of AD incidence indicate that the predictive value of short rLTL among non-carriers is higher when measured earlier in the prodromal phase, as indicated by the significant time-interaction in the statistical model and the plotted cumulative incidence curve. Another result reinforcing short LTL as a predictive marker for participants not carrying the *APOE* ε4 allele is its predictive ability for AD incidence over and above commonly available lifestyle risk markers previously associated with neurodegenerative disorders, which we discuss below.

Although differential predictive power of LTL for *APOE* ε4-carriers and non-carriers was expected, the different directions of associations in this study were not. Prior studies have observed differential LTL-effects for dementia and age-related cognitive dysfunction for ε4-carriers and non-carriers [26, 29–32], but our study is the first to observe a *stronger* predictive effect in non-carriers. Given that LTL is a non-specific biomarker associated with multiple processes in the body, such as oxidative stress, inflammation, immune function, cardiovascular function [7, 8, 55], any mechanistic proposals on the relationship between *APOE* ε4 carriage and LTL in AD etiology would be premature based on the present observational findings. With that said, evidence for potentially differential disease mechanisms for AD in *APOE* ε4-carriers and non-carriers has been reported [1, 56, 57], and it cannot be ruled out that LTL is associated with a different mechanistic pathway in non-carriers than in carriers. For instance, gene expression analyses have identified modules of genes related to immunological and cardiovascular pathways to be expressed in AD brain samples of *APOE* ε4 non-carriers [57], processes which have also been linked to LTL [7, 8, 55]. In contrast, LTL may be relatively less related to neuropathological processes shown to be accelerated in *APOE* ε4-carriers [1, 4, 56, 58], such as neuronal amyloid-β and tau deposition, blood-brain barrier dysfunction, or neuronal atrophy (but see [59–61]). *APOE* ε4-carriers and non-carriers have also been shown to be differentially represented in identified subcategories of AD [62, 63], reinforcing the notion of potentially differential disease mechanisms. However, more research into how LTL relates to different AD-related disease mechanisms and disease heterogeneity is warranted to gain a better understanding of the mechanistic basis of the present findings.

Both the Fine-Gray and the cause-specific hazard models showed similar association profiles between LTL and AD, as well as for VaD and death. The cause-specific hazard model for AD among *APOE* ε4 non-carriers with short rLTL did not however reach conventional levels of significance (*P* = 0.07). As the two models reflect different types of hazard functions, their combined use has been advocated to reach a more complete understanding of the associations [37–39]. The differences in findings across models should not be seen as surprising and are merely a result of considering different risk sets. Specifically, the cause-specific hazard model reflects covariate effects on the instantaneous rate of occurrence in individuals who are currently alive and dementia-free, whereas the Fine-Gray model reflects covariate effects across all participants who have not experienced the event-of-interest at the time. Furthermore, the two models serve complementary purposes in that cause-specific hazard models are considered more appropriate for estimating etiological associations between covariates and the event, while the Fine-Gray model is considered more appropriate for estimating incidence or predicting prognosis [37, 40]. Our findings support the use of the Fine-Gray and cause-specific models side-by-side, to obtain a more complete understanding of covariates effect on AD in the presence of competing risks. Taken together, our results suggest that short LTL may be a valuable predictor or biomarker of the AD incidence in non-carriers of the *APOE* *ε4* allele, but the association does not answer the question of whether LTL is mechanistically contributing to AD etiology or it is merely a predictive AD biomarker.

Significant non-linearity of the LTL association with AD was evidenced in our study, as showed by the spline analyses used to estimate the best fitting shape of covariate associations in a model [51]. Accordingly, both short and long LTL were associated with increased incidence and cause-specific risk of AD among non-*APOE* ε4-carriers (sHR and csHR > 1), although the effect for longer than average LTL was not significant. This was reinforced by the profiles shown in the cumulative incidence and csHR plots. A similar profile can be observed for the LTL-VaD sHR and csHR, although non-significant. For *APOE* ε4-carriers, short and long rLTLs showed a trend of decreased incidence and cause-specific risk of AD, when compared with medium rLTL *APOE* ε4-carriers. Again, these opposing patterns could be indicative of LTL being associated with different disease pathways in carriers and non-carriers. Similar opposing effects of short TL was seen in an experimental rodent study, where TL shortening reduced amyloid plaque pathology and cognitive deficits in the AD mouse model, whereas it was associated with poorer neurocognitive outcomes in the non-AD mouse [64]. Previous studies on amnestic mild cognitive impairment and AD also converge with our findings on non-linear associations between TL and neurocognitive outcomes, with short and long LTL being associated with increased disease risk [26, 27]. In contrast, the observed linear association profile of rLTL with death, with increased (sHR and csHR > 1) association with short rLTL and decreased (sHR and csHR < 1) with long rLTL, are in accordance with relevant mortality studies [10, 12, 13]. LTL associations with AD or other dementia disorders may have gone undetected in previous studies where the association was assumed to be monotonic. Thus, our results highlight the importance of testing for potential non-linearities in LTL-dementia associations.

We showed here that LTL was predictive of AD incidence in non-*APOE* ε4-carriers, over and above a large set of commonly available markers modifiable by environmental factors and lifestyle. The sizable number of lifestyle-related risk factors and other relevant covariates selected for our analyses is one of the strengths of our study, being greater than prior time-to-event studies for AD or dementia prediction [10, 24–26, 28]. Our covariate effects largely replicate previous literature findings and thereby further validate our results. For instance, the association of blood cholesterols with increased AD incidence is in accordance with the well-known association with AD risk [35]. The weak association of plasma glucose with a decreased incidence of AD could reflect the previously established relationship of hypoglycemia, malnutrition, muscle weight loss, and low BMI with AD progression, especially in older cohorts [36, 65]. Also, our findings reinforce previous knowledge that being female increases the incidence and the cause-specific risk of AD [1, 66]. A full discussion of covariate effects is beyond the scope of the paper, as complex covariate associations may arise across the two statistical models employed, for the abovementioned reasons. This is particularly evidenced by the opposing associations of *APOE* ε4 carriage (or plasma glucose) with death in the two models. In the Fine-Gray model, we found a strong positive association between *APOE* ε4-carriers and AD. This is expected and further explains the opposing negative association between *APOE* ε4-carriers and deaths. In this model, all demented, and therefore more *APOE* ε4-carriers, are at risk to progress to death, hence, creating a hypothetical overall population at risk. Since the model does not consider death with dementia, the higher proportion of *APOE* ε4-carriers among the “immortal” dementia cases in this risk population causes an apparent decrease in the sHR of death for *APOE*. Nevertheless, the results are still valid for estimating the incidence for *APOE* ε4-carriers and describe the predictive performance of the covariate. In contrast, the cause-specific hazard model estimates the risk of death for *APOE* ε4-carriers among healthy and non-demented participants. By excluding those who have progressed to dementia, the model considers a more narrow population at risk and avoids the influence of “immortals” in the risk estimation. However, this risk set is not valid for estimating incidences in the overall population [37, 67].

We did not observe an association between LTL and VaD in our study sample, analyzed as a competing risk event for AD in our models. Although this could be due to the lower power for the relatively smaller subset of VaD cases, in combination with the null effects for the all-cause dementia sensitivity analyses (Supplementary Table 3), the pattern of findings suggests that LTL may be specifically related with AD prediction. There are strong a priori reasons to hypothesize an LTL-VaD relationship, given the strong association between short LTL and cardiovascular disease [55]; however, prior studies are scarce and show inconsistent findings. Evidence from a case-control study indicated that VaD cases have short LTL [68] while a similar study found no evidence [20]. Evidence of LTL’s role as a VaD predictor is even more limited, but one time-to-event analysis study showed a weak association of short LTL with increased VaD incidence [25]. Thus, the value of LTL for the prediction of VaD remains to be elucidated. VaD has a multifactorial etiology, with a less clear genetic background than AD; therefore, its association with lifestyle risk factors as hypertension, inflammation, and obesity that leads to cerebrovascular disease are expected to stand out as predictors [35, 50, 68, 69]. Some of our covariate associations, such as the effects of pulse pressure and erythrocyte sedimentation rate on increased VaD hazards are in accordance with the abovementioned expectations. Also, the observed association of male gender with increased VaD incidence may reflect the higher prevalence of cerebrovascular disease among males [50]. Importantly, our time-to-event models evidenced a different set of covariates with significant effects on VaD and AD, validating our clinical differential diagnosis of dementia subtypes.

### Limitations

A limitation of this study is the lack of a neuropathologically confirmed dementia diagnoses. Nevertheless, the diagnostic procedure was comprehensive, considering long-term medical documentation from multiple clinical disciplines combined with health and cognitive assessments, and clear differences in observed covariate associations were observed for the AD and VaD categories, reinforcing their validity. Although caution should be exercised in considering the significance threshold as P < 0.05, the careful employment of two complementary time-to-event models strengthens the validity of our findings and follows a recommendation of prior authors [38, 39]. Nevertheless, replication of the present findings in larger samples is desirable. Furthermore, some selection bias could have been present in our data because some participants with short LTL may have died before study enrolment, or become demented and thereby fulfilled study exclusion criteria. Such biases likely lead to an underestimation of the LTL-AD association, but should not invalidate the significant effects that we did observe.

## Conclusions

Our findings indicate that short LTL may be a valuable predictor of AD for the non-carriers of the *APOE* ε4 allele, who constitute up to half of the AD cases. The present findings also highlight the importance of accounting for competing risks of mortality and other dementia types, as well as non-linearities in LTL associations with dementia pathogeneses and outcomes. More generally, improved knowledge of the type of genotype-biomarker interactions observed here is highly relevant for personalized prediction strategies, an important subgoal of personalized medicine. In the long run, advances in genome technology and more accessible costs for genome analyses may enable the combination of genotype and LTL measurements to be used in routine risk assessment for AD.

### Supplementary Information

**Additional file 1: Supplementary Fig 1.** Flowchart of the studied population. **Supplementary Table 1**. Descriptive characteristics of the rLTL tertiles. **Supplementary Table 2.** Fine-Gray and cause-specific hazard models with medium telomere as reference group. **Supplementary Table 3.** Fine-Gray and cause-specific hazard models predicting the risk of all-cause dementia. **Supplementary Fig 2.** Non-linear association between residualized leukocyte telomere length (rLTL) and the risk of Alzheimer’s disease (AD), investigated by natural splines. **Supplementary Fig 3.** Area under the receiver operating characteristic (ROC) curve (AUC) over the study time-course, showing the prediction ability of the models for Alzheimer’s disease (AD). **Supplementary Fig 4.** Area under the receiver operating characteristic (ROC) curve (AUC) over the study time-course, showing the increase in prediction ability for Alzheimer’s disease (AD) by residualized leukocyte telomere length (rLTL) in non-apolipoprotein (*APOE*) ε4-carriers. **Models’ equations.** Equations of the subdistribution (Fine-Gray) hazard function, cause-specific hazard function, and cumulative incidence function (CIF) in the presence of competing risks.

## Data Availability

The datasets used and/or analyzed during the current study are available from the corresponding author on reasonable request, as long as the data transfer is in agreement with the European Union legislation on the General Data Protection Regulation and Umeå University data protection policies.

## Abbreviations

AD: Alzheimer’s disease
AIC: Akaike information criteria
*APOE* ε4: Apolipoprotein E ε4
BIC: Bayesian information criteria
CI: Confidence interval
CIF: Cumulative incidence function
HBB: Hemoglobin subunit beta
csHR: Cause-specific hazard ratio
LTL: Leukocyte telomere length
PCR: Polymerase chain reaction
rLTL: Residualized leukocyte telomere length
sHR: Subdistribution hazard ratio
TEL: Telomere
VaD: Vascular dementia

## References

[1] Yamazaki Y, Zhao N, Caulfield TR, Liu CC, Bu GJ (2019). Apolipoprotein E and Alzheimer disease: pathobiology and targeting strategies. Nat Rev Neurol..

[2] James BD, Bennett DA (2019). Causes and patterns of dementia: an update in the era of redefining Alzheimer's disease. Annu Rev Public Health..

[3] Aisen PS, Cummings J, Jack CR, Morris JC, Sperling R, Frolich L (2017). On the path to 2025: understanding the Alzheimer’s disease continuum. Alzheimer’s Res Ther..

[4] Serrano-Pozo A, Das S, Hyman BT (2021). APOE and Alzheimer’s disease: advances in genetics, pathophysiology, and therapeutic approaches. Lancet Neurol..

[5] Crean S, Ward A, Mercaldi CJ, Collins JM, Cook MN, Baker NL, Arrighi HM (2011). Apolipoprotein E epsilon 4 prevalence in Alzheimer’s disease patients varies across global populations: a systematic literature review and meta-analysis. Dement Geriatr Cogn Disord..

[6] Cuyvers E, Sleegers K (2016). Genetic variations underlying Alzheimer’s disease: evidence from genome-wide association studies and beyond. Lancet Neurol..

[7] Aubert G, Lansdorp PM (2008). Telomeres and aging. Physiol Rev..

[8] Blackburn EH, Epel ES, Lin J (2015). Human telomere biology: a contributory and interactive factor in aging, disease risks, and protection. Science..

[9] O'Sullivan RJ, Karlseder J (2010). Telomeres: protecting chromosomes against genome instability. Nat Rev Mol Cell Biol..

[10] Honig LS, Kang MS, Schupf N, Lee JH, Mayeux R (2012). Association of shorter leukocyte telomere repeat length with dementia and mortality. Arch Neurol..

[11] Lapham K, Kvale MN, Lin J, Connell S, Croen LA, Dispensa BP, Fang L, Hesselson S, Hoffmann TJ, Iribarren C, Jorgenson E, Kushi LH, Ludwig D, Matsuguchi T, McGuire WB, Miles S, Quesenberry CP, Rowell S, Sadler M, Sakoda LC, Smethurst D, Somkin CP, van den Eeden SK, Walter L, Whitmer RA, Kwok PY, Risch N, Schaefer C, Blackburn EH (2015). Automated assay of telomere length measurement and informatics for 100,000 subjects in the genetic epidemiology research on adult health and aging (GERA) cohort. Genetics..

[12] Arbeev KG, Verhulst S, Steenstrup T, Kark JD, Bagley O, Kooperberg C, Reiner AP, Hwang SJ, Levy D, Fitzpatrick AL, Christensen K, Yashin AI, Aviv A (2020). Association of leukocyte telomere length with mortality among adult participants in 3 longitudinal studies. JAMA..

[13] Wang Q, Zhan YQ, Pedersen NL, Fang F, Hagg S (2018). Telomere length and all-cause mortality: a meta-analysis. Ageing Res Rev..

[14] Aviv A, Shay JW (2018). Reflections on telomere dynamics and ageing-related diseases in humans. Philos Trans R Soc B-Biol Sci..

[15] Boccardi V, Arosio B, Cari L, Bastiani P, Scamosci M, Casati M, Ferri E, Bertagnoli L, Ciccone S, Rossi PD, Nocentini G, Mecocci P (2020). Beta-carotene, telomerase activity and Alzheimer’s disease in old age subjects. Eur J Nutr..

[16] Forero DA, Gonzalez-Giraldo Y, Lopez-Quintero C, Castro-Vega LJ, Barreto GE, Perry G (2016). Meta-analysis of telomere length in Alzheimer’s disease. J Gerontol A Biol Sci Med Sci..

[17] Honig LS, Schupf N, Lee JH, Tang MX, Mayeux R (2006). Shorter telomeres are associated with mortality in those with APOE epsilon 4 and dementia. Ann Neurol..

[18] Panossian LA, Porter VR, Valenzuela HF, Zhu X, Reback E, Masterman D, Cummings JL, Effros RB (2003). Telomere shortening in T cells correlates with Alzheimer’s disease status. Neurobiol Aging..

[19] Scarabino D, Broggio E, Gambina G, Corbo RM (2017). Leukocyte telomere length in mild cognitive impairment and Alzheimer’s disease patients. Exp Gerontol..

[20] Zekry D, Herrmann FR, Irminger-Finger I, Graf C, Genet C, Vitale AM, Michel JP, Gold G, Krause KH (2010). Telomere length and ApoE polymorphism in mild cognitive impairment, degenerative and vascular dementia. J Neurol Sci..

[21] Zekry D, Herrmann FR, Irminger-Finger I, Ortolan L, Genet C, Vitale AM, Michel JP, Gold G, Krause KH (2010). Telomere length is not predictive of dementia or MCI conversion in the oldest old. Neurobiol Aging..

[22] Takata Y, Kikukawa M, Hanyu H, Koyama S, Shimizu S, Umahara T, Sakurai H, Iwamoto T, Ohyashiki K, Ohyashiki JH (2012). Association between ApoE phenotypes and telomere erosion in Alzheimer’s disease. J Gerontol A Biol Sci Med Sci..

[23] Moverare-Skrtic S, Johansson P, Mattsson N, Hansson O, Wallin A, Johansson JO (2012). Leukocyte telomere length (LTL) is reduced in stable mild cognitive impairment but low LTL is not associated with conversion to Alzheimer’s disease: a pilot study. Exp Gerontol..

[24] Koh SH, Choi SH, Jeong JH, Jang JW, Park KW, Kim EJ, et al. Telomere shortening reflecting physical aging is associated with cognitive decline and dementia conversion in mild cognitive impairment due to Alzheimer's disease. Aging (Albany NY). 2020. 10.18632/aging.102893. 10.18632/aging.102893 PMC7093181 32126022

[25] Hinterberger M, Fischer P, Huber K, Krugluger W, Zehetmayer S (2017). Leukocyte telomere length is linked to vascular risk factors not to Alzheimer’s disease in the VITA study. J Neural Transm (Vienna)..

[26] Fani L, Hilal S, Sedaghat S, Broer L, Licher S, Arp PP, van Meurs JBJ, Ikram MK, Ikram MA (2020). Telomere length and the risk of Alzheimer’s disease: the Rotterdam study. J Alzheimers Dis..

[27] Roberts RO, Boardman LA, Cha RH, Pankratz VS, Johnson RA, Druliner BR, Christianson TJH, Roberts LR, Petersen RC (2014). Short and long telomeres increase risk of amnestic mild cognitive impairment. Mech Ageing Dev..

[28] Martin-Ruiz C, Dickinson HO, Keys B, Rowan E, Kenny RA, von Zglinicki T (2006). Telomere length predicts poststroke mortality, dementia, and cognitive decline. Ann Neurol..

[29] Hagg S, Zhan Y, Karlsson R, Gerritsen L, Ploner A, van der Lee SJ (2017). Short telomere length is associated with impaired cognitive performance in European ancestry cohorts. Transl Psychiatry..

[30] Mahoney E, Dumitrescu L, Seto M, Nudelman K, Buckley R, Gifford K (2019). Telomere length associations with cognition depend on Alzheimer’s disease biomarkers. Alzheimers Dement (NY)..

[31] Wikgren M, Karlsson T, Nilbrink T, Nordfjall K, Hultdin J, Sleegers K (2012). APOE epsilon 4 is associated with longer telomeres, and longer telomeres among epsilon 4 carriers predicts worse episodic memory. Neurobiol Aging..

[32] Yaffe K, Lindquist K, Kluse M, Cawthon R, Harris T, Hsueh WC, Simonsick EM, Kuller L, Li R, Ayonayon HN, Rubin SM, Cummings SR, Health ABC Study (2011). Telomere length and cognitive function in community-dwelling elders: findings from the Health ABC Study. Neurobiol Aging..

[33] Nyberg L, Boraxbekk CJ, Sorman DE, Hansson P, Herlitz A, Kauppi K (2020). Biological and environmental predictors of heterogeneity in neurocognitive ageing Evidence from Betula and other longitudinal studies. Ageing Res Rev..

[34] Nilsson LG, Backman L, Erngrund K, Nyberg L, Adolfsson R, Bucht G (1997). The Betula prospective cohort study: memory, health and aging. Aging Neuropsychol Cogn..

[35] Andrews SJ, Fulton-Howard B, O'Reilly P, Marcora E, Goate AM (2021). Causal associations between modifiable risk factors and the Alzheimer’s phenome. Ann Neurol..

[36] Lee H, Kim K, Lee YC, Kim S, Won HH, Yu TY, Lee EM, Kang JM, Lewis M, Kim DK, Myung W (2020). Associations between vascular risk factors and subsequent Alzheimer’s disease in older adults. Alzheimers Res Ther..

[37] Austin PC, Lee DS, Fine JP (2016). Introduction to the analysis of survival data in the presence of competing risks. Circulation..

[38] Latouche A, Allignol A, Beyersmann J, Labopin M, Fine JP (2013). A competing risks analysis should report results on all cause-specific hazards and cumulative incidence functions. J Clin Epidemiol..

[39] Varadhan R, Weiss CO, Segal JB, Wu AW, Scharfstein D, Boyd C (2010). Evaluating health outcomes in the presence of competing risks a review of statistical methods and clinical applications. Med Care..

[40] Austin PC, Fine JP (2017). Practical recommendations for reporting Fine-Gray model analyses for competing risk data. Stat Med..

[41] Fine JP, Gray RJ (1999). A proportional hazards model for the subdistribution of a competing risk. J Am Stat Assoc..

[42] Nilsson LG, Adolfsson R, Backman L, de Frias CM, Molander B, Nyberg L (2004). Betula: A prospective cohort study on memory, health and aging. Aging Neuropsychol Cogn..

[43] American Psychiatric Association (2000). Diagnostic and statistical manual of mental disorders-IV-TR.

[44] Medlineplus.gov: Neurodegenerative diseases. https://medlineplus.gov/degenerativenervediseases.html. Bethesda, EUA. 2020. Accessed 20 Feb 2021.

[45] Reiman EM, Arboleda-Velasquez JF, Quiroz YT, Huentelman MJ, Beach TG, Caselli RJ (2020). Exceptionally low likelihood of Alzheimer’s dementia in APOE2 homozygotes from a 5,000-person neuropathological study. Nat Commun..

[46] Cawthon RM (2002). Telomere measurement by quantitative PCR. Nucleic Acids Res..

[47] Nordfjall K, Osterman P, Melander O, Nilsson P, Roos G (2007). hTERT T-1327/C polymorphism is not associated with age-related telomere attrition in peripheral blood. Biochem Biophys Res Commun..

[48] Pudas S, Josefsson M, Adolfsson A, Landfors M, Kauppi K, Veng-Taasti L (2020). Short leukocyte telomeres, but not telomere attrition rates, predict memory decline in the 20-year longitudinal betula study. J Gerontol A Biol Sci Med Sci..

[49] Nilsson LG, Adolfsson R, Backman L, Cruts M, Nyberg L, Small BJ, Van Broeckhoven C (2006). The influence of APOE status on episodic and semantic memory: data from a population-based study. Neuropsychology..

[50] Virani SS, Alonso A, Benjamin EJ, Bittencourt MS, Callaway CW, Carson AP, Chamberlain AM, Chang AR, Cheng S, Delling FN, Djousse L, Elkind MSV, Ferguson JF, Fornage M, Khan SS, Kissela BM, Knutson KL, Kwan TW, Lackland DT, Lewis TT, Lichtman JH, Longenecker CT, Loop MS, Lutsey PL, Martin SS, Matsushita K, Moran AE, Mussolino ME, Perak AM, Rosamond WD, Roth GA, Sampson UKA, Satou GM, Schroeder EB, Shah SH, Shay CM, Spartano NL, Stokes A, Tirschwell DL, VanWagner LB, Tsao CW, On behalf of the American Heart Association Council on Epidemiology and Prevention Statistics Committee and Stroke Statistics Subcommittee (2020). Heart disease and stroke statistics-2020 update: a report from the American Heart Association. Circulation..

[51] Gauthier J, Wu QV, Gooley TA (2020). Cubic splines to model relationships between continuous variables and outcomes: a guide for clinicians. Bone Marrow Transplant..

[52] Zhang ZH. Survival analysis in the presence of competing risks. Ann Transl Med. 2017. 10.21037/atm.2016.08.62. 10.21037/atm.2016.08.62 PMC5326634 28251126

[53] Zhang ZH, Cortese G, Combescure C, Marshall R, Lee M, Lim HJ, et al. Overview of model validation for survival regression model with competing risks using melanoma study data. Ann Transl Med. 2018. 10.21037/atm.2018.07.38. 10.21037/atm.2018.07.38 PMC6186983 30364028

[54] Wikgren M, Karlsson T, Lind J, Nilbrink T, Hultdin J, Sleegers K, et al. Longer leukocyte telomere length is associated with smaller hippocampal volume among non-demented APOE epsilon 3/epsilon 3 subjects. Plos One. 2012;7(4). 10.1371/journal.pone.0034292. 10.1371/journal.pone.0034292 PMC3323621 22506016

[55] Spyridopoulos I, von Zglinicki T. Telomere length predicts cardiovascular disease. BMJ. 2014;349(jul08 19). 10.1136/bmj.g4373. 10.1136/bmj.g4373 25005709

[56] Emrani S, Arain HA, DeMarshall C, Nuriel T (2020). APOE4 is associated with cognitive and pathological heterogeneity in patients with Alzheimer’s disease: a systematic review. Alzheimers Res Ther..

[57] Jiang S, Tang L, Zhao N, Yang WL, Qiu Y, Chen HZ. A systems view of the differences between APOE epsilon 4 carriers and non-carriers in Alzheimer’s disease. Front Aging Neurosci. 2016;8. 10.3389/fnagi.2016.00171. 10.3389/fnagi.2016.00171 PMC4941795 27462267

[58] Long JM, Holtzman DM (2019). Alzheimer disease: an update on pathobiology and treatment strategies. Cell..

[59] Raj DDA, Moser J, van der Pol SMA, van Os RP, Holtman IR, Brouwer N (2015). Enhanced microglial pro-inflammatory response to lipopolysaccharide correlates with brain infiltration and blood-brain barrier dysregulation in a mouse model of telomere shortening. Aging Cell..

[60] King KS, Kozlitina J, Rosenberg RN, Peshock RM, McColl RW, Garcia CK (2014). Effect of leukocyte telomere length on total and regional brain volumes in a large population-based cohort. JAMA Neurol..

[61] Jacobs EG, Epel ES, Lin J, Blackburn EH, Rasgon NL (2014). Relationship between leukocyte telomere length, telomerase activity, and hippocampal volume in early aging. JAMA Neurol..

[62] Ferreira D, Nordberg A, Westman E (2020). Biological subtypes of Alzheimer disease: a systematic review and meta-analysis. Neurology..

[63] Mukherjee S, Mez J, Trittschuh E, Saykin A, Gibbons L, Fardo D (2020). Genetic data and cognitively defined late-onset Alzheimer’s disease subgroups. Mol Psychiatry..

[64] Rolyan H, Scheffold A, Heinrich A, Begus-Nahrmann Y, Langkopf BH, Holter SM (2011). Telomere shortening reduces Alzheimer’s disease amyloid pathology in mice. Brain..

[65] Jimenez A, Pegueroles J, Carmona-Iragui M, Vilaplana E, Montal V, Alcolea D, et al. Weight loss in the healthy elderly might be a non-cognitive sign of preclinical Alzheimer’s disease. Oncotarget. 2017. 10.18632/oncotarget.22218. 10.18632/oncotarget.22218 PMC5739594 29285207

[66] Frigerio CS, Wolfs L, Fattorelli N, Thrupp N, Voytyukt I, Schmidt I (2019). The major risk factors for Alzheimer’s disease: age, sex, and genes modulate the microglia response to A-beta plaques. Cell Rep..

[67] Wolters FJ, Yang Q, Biggs ML, Jakobsdottir J, Li S, Evans DS, Bis JC, Harris TB, Vasan RS, Zilhao NR, Ghanbari M, Ikram MA, Launer L, Psaty BM, Tranah GJ, Kulminski AM, Gudnason V, Seshadri S, for the E2-CHARGE investigators (2019). The impact of APOE genotype on survival: results of 38,537 participants from six population-based cohorts (E2-CHARGE). Plos One..

[68] von Zglinicki T, Serra V, Lorenz M, Saretzki G, Lenzen-Grossimlighaus R, Gessner R (2000). Short telomeres in patients with vascular dementia: an indicator of low antioxidative capacity and a possible risk factor?. Lab Invest..

[69] Ikram MA, Bersano A, Manso-Calderon R, Jia JP, Schmidt H, Middleton L (2017). Genetics of vascular dementia - review from the ICVD working group. BMC Med..

