# Correction: Short leukocyte telomeres predict 25-year Alzheimer’s disease incidence in non-*APOE* ε4-carriers

**DOI:** 10.1186/s13195-024-01388-w

**Published:** 2024-02-16

**Authors:** Fernanda Schäfer Hackenhaar, Maria Josefsson, Annelie Nordin Adolfsson, Mattias Landfors, Karolina Kauppi, Magnus Hultdin, Rolf Adolfsson, Sofie Degerman, Sara Pudas

**Affiliations:** 1 Department of Integrative Medical Biology, Umeå University, Umeå, SE-901 87 Sweden; 2 Umeå Center for Functional Brain Imaging, Umeå University, Umeå, SE-90 187 Sweden; 3 Department of Statistics, USBE, Umeå University, Umeå, SE-901 87 Sweden; 4 Center for Ageing and Demographic Research, Umeå University, Umeå, SE-901 87 Sweden; 5 Department of Clinical Sciences, Umeå University, Umeå, SE-901 85 Sweden; 6 Department of Medical Biosciences, Pathology, Umeå University, SE-901 85 Umeå, Sweden; 7 Department of Medical Epidemiology and Biostatistics, Karolinska Institute, Stockholm, SE-171 77 Sweden; 8 Department of Clinical Microbiology, Umeå University, Umeå, SE-901 85 Sweden

**Correction: Alz Res Therapy 13, 130 (2021)**

**https://doi.org/10.1186/s13195-021-00871-y**

Following the publication of the original article [1], the authors discovered that in contrast to what is stated in the article, the variable residualized leukocyte telomere length (rLTL) was not residualized for age, only gender. However, as the article describes, the effect of age and quadratic age were still accounted for by including these variables into the statistical models, as is common practice in the field. The results and conclusions reported in the paper remain the same, although potential residual confounding between rLTL tertile groups and chronological age must be considered when interpreting the findings. Future studies with narrow age-cohort designs will be needed to confirm the reported results.

The original article [1] has been updated.
