# Supplementary material for: Short leukocyte telomeres predict 25-year Alzheimer's disease incidence in non-APOE ε4-carriers
Source: Alzheimers Res Ther. 2021 Jul 15;13:130. doi: 10.1186/s13195-021-00871-y (PMC8283833; doi:10.1186/s13195-021-00871-y)
Supplement: Supplementary file 1 — Additional file 1: Supplementary Fig 1. Flowchart of the studied population. Supplementary Table 1. Descriptive characteristics of the rLTL tertiles. Supplementary Table 2. Fine-Gray and cause-specific hazard models with medium telomere as reference group. Supplementary Table 3. Fine-Gray and cause-specific hazard models predicting the risk of all-cause dementia. Supplementary Fig 2. Non-linear association between residualized leukocyte telomere length (rLTL) and the risk of Alzheimer’s disease (AD), investigated by natural splines. Supplementary Fig 3. Area under the receiver operating characteristic (ROC) curve (AUC) over the study time-course, showing the prediction ability of the models for Alzheimer’s disease (AD). Supplementary Fig 4. Area under the receiver operating characteristic (ROC) curve (AUC) over the study time-course, showing the increase in prediction ability for Alzheimer’s disease (AD) by residualized leukocyte telomere length (rLTL) in non-apolipoprotein (APOE) ε4-carriers. Models’ equations. Equations of the subdistribution (Fine-Gray) hazard function, cause-specific hazard function, and cumulative incidence function (CIF) in the presence of competing risks. [file 13195_2021_871_MOESM1_ESM.pdf]

Additional files

Studied population

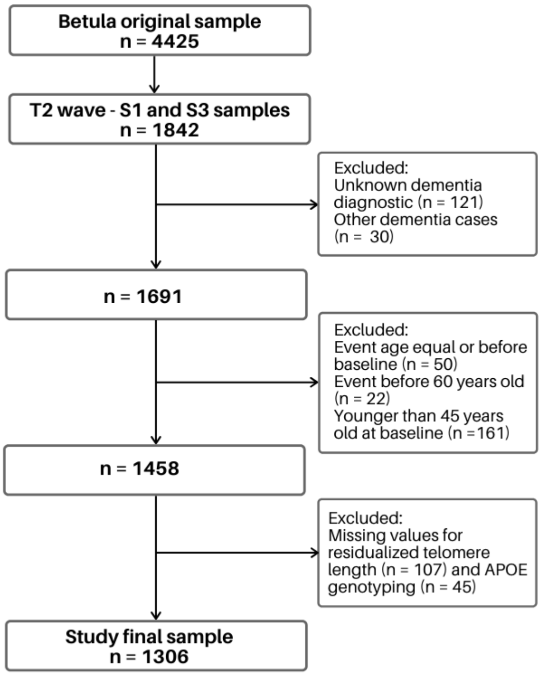

Supplementary Fig 1. Flowchart of the studied population.

Descriptives for rLTL tertiles

Supplementary Table 1. Descriptive characteristics of the rLTL tertiles

	Short rLTL (tertile 1)	Medium rLTL (tertile 2)	Long rLTL (tertile 3)
Number	439	436	431
APOE ε4-carriers	127 (28.9%)	100 (22.9%)	136 (31.6%)
Non-*APOE* ε4-carriers	312 (71.1%)	336 (77.1%)	295 (68.4%)
AD	61 (13.9%)	45 (10.3%)	43 (10%)
AD carriers/non-carriers	28/33	26/19	24/19
VaD	40 (9.1%)	28 (6.4%)	28 (6.5%)
Deceased non-demented	207 (47.2%)	156 (35.8%)	102 (23.6%)
Healthy	131 (29.8%)	207 (47.5%)	258 (59.9%)

AD, Alzheimer's disease; *APOE* ε4, apolipoprotein E ε4; rLTL, residualized leukocyte telomere length; VaD, vascular dementia.

Fine-Gray and cause-specific hazard models with medium telomere as reference group

Supplementary Table 2. Fine-Gray and cause-specific hazard models for Alzheimer's disease

	sHR	*P*-value	csHR	*P*-value
Non-*APOE* ε4-carriers with medium rLTL	1 (reference)	1 (reference)	1 (reference)	1 (reference)
Non-*APOE* ε4-carriers with short rLTL	2.40	0.03 *	1.67	0.07
Non-*APOE* ε4-carriers with long rLTL	1.42	0.28	1.38	0.32
APOE ε4-carriers with medium rLTL	1 (reference)	1 (reference)	1 (reference)	1 (reference)
APOE ε4-carriers with short rLTL	0.61	0.08	0.72	0.24
APOE ε4-carriers with long rLTL	0.57	0.05	0.57	0.05

Models with dummy-coding the short, medium and long rLTL groups among *APOE* ε4-carriers and non-carriers into six groups. Two different models with the same variables were employed, one with non-*APOE* ε4-carriers with medium rLTL as the reference group, and another with *APOE* ε4-carriers with medium rLTL as the reference group. All models were adjusted by high cholesterol, pulse pressure, plasma glucose, erythrocyte sedimentation rate, lymphocyte proportion, age and age squared. Time-interaction for short telomere and lymphocyte proportion in non-*APOE* ε4-carriers were included for the Fine-Gray model. *APOE* ε4, apolipoprotein E ε4; csHR, cause-specific hazard ratio of cause-specific hazard model; rLTL, residualized leukocyte telomere length; sHR, ratio of the subdistribution hazards of Fine-Gray model. Time from baseline, in years, was used as time scale. (Total n = 1306, Alzheimer's disease n = 149). * $P < 0.05$.

All-cause dementia Fine-Gray and cause-specific hazard models

Supplementary Table 3. Fine-Gray and cause-specific hazard models predicting the risk of all-cause dementia (n = 1306)

	Fine-Gray model<br>[sHR (95% CI); *P*-value]	Cause-specific hazard model<br>[csHR (95% CI); *P*-value]
	All-cause dementia (n = 245)	
Short residualized telomere length	0.99 (0.725–1.349); *P* = 0.94	1.16 (0.856–1.587); *P* = 0.33
Long residualized telomere length	1.02 (0.737–1.420); *P* = 0.89	0.97 (0.693–1.349); *P* = 0.84
Apolipoprotein E ε4-carriers	2.56 (1.975–3.331); *P* < 0.0001	2.64 (2.035–3.417); *P* < 0.0001
High cholesterol (≥ 240 mg/dL)	1.40 (1.029–1.917); *P* = 0.03	1.37 (0.973–1.780); *P* = 0.07
Pulse pressure, mmHg	1.00 (0.997–1.012); *P* = 0.21	1.01 (1.001–1.016); *P* = 0.03
Plasma glucose, mg/dL	0.99 (0.989–1.001); *P* = 0.11	0.998 (0.994–1.003); *P* = 0.47
Sedimentation rate, mm/h	0.99 (0.987–1.010); *P* = 0.85	1.00 (0.994–1.019); *P* = 0.27
Lymphocyte proportion	0.30 (0.053–1.739); *P* = 0.18	0.21 (0.036–1.204); *P* = 0.07
Gender, male	0.56 (0.419–0.747); *P* < 0.0001	0.68 (0.511–0.912); *P* = 0.009
Age at baseline, years	2.71 (2.107–3.499); *P* < 0.0001	2.33 (1.797–3.029); *P* < 0.0001
Age squared	0.993 (0.992–0.995); *P* < 0.0001	0.995 (0.993–0.996); *P* < 0.0001
	Pseudo likelihood ratio = 320	Akaike information criteria = 2937

CI, confidence interval; csHR, cause-specific hazard ratio of cause-specific hazard model, accounting for competing risks; sHR, ratio of the subdistribution hazards of Fine-Gray model. Time from baseline, in years, was used as the time scale. There were no significant rLTL vs. apolipoprotein E ε4 interactions or time-interactions in the all-cause dementia models.

Non-linear association between rLTL and AD risk

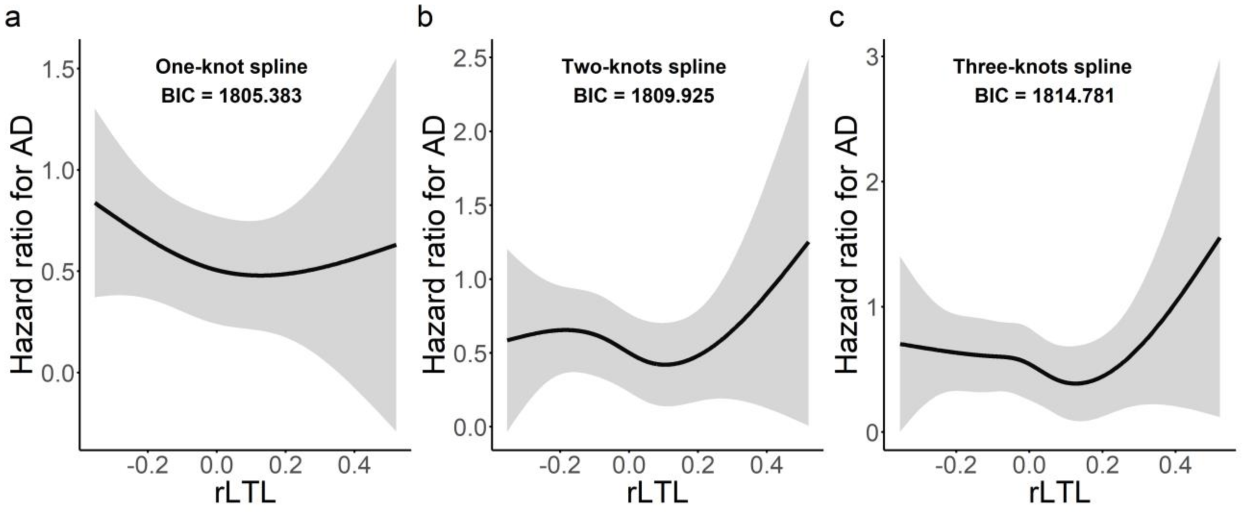

Supplementary Fig 2. The non-linear association between residualized leukocyte telomere length (rLTL) and the risk of Alzheimer's disease (AD) was investigated by including (**a**) one-knot, (**b**) two-knots, and (**c**) three-knots natural splines. The cause-specific hazard models were estimated for a representative female non-apolipoprotein (*APOE*) ε4-carrier and normal cholesterol levels, with median values of pulse pressure, plasma glucose, erythrocyte sedimentation rate, lymphocyte proportion, and age squared. The lowest Bayesian information criteria (BIC) was used to identify the best-fitted model.

Models' validation

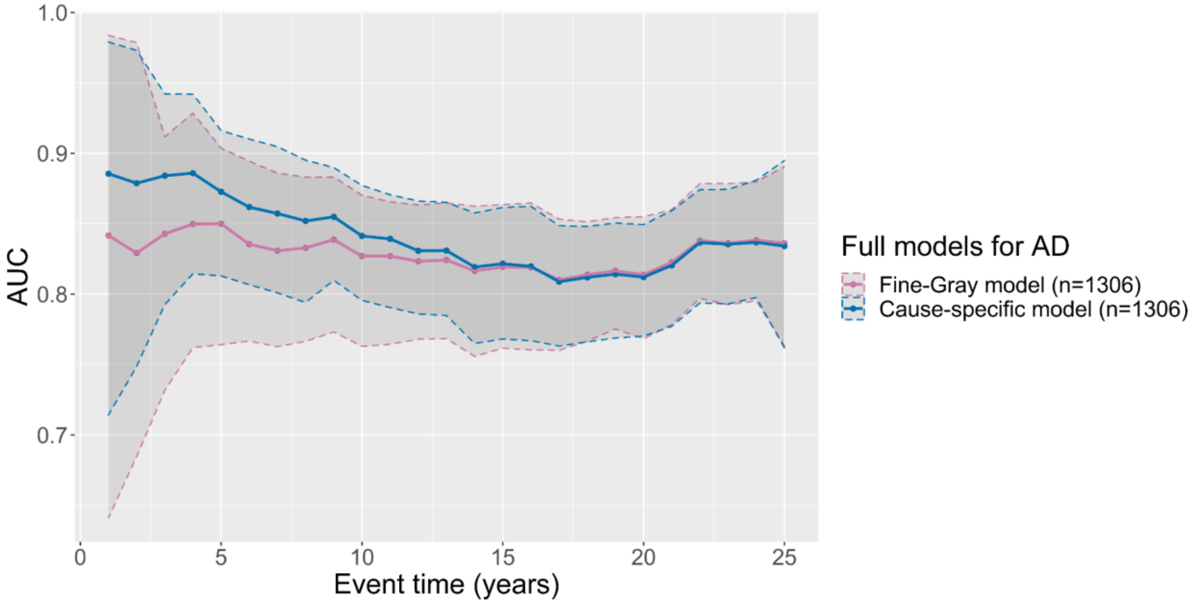

Supplementary Fig 3. Area under the receiver operating characteristic (ROC) curve (AUC) over the study time-course, showing the prediction ability of the models for Alzheimer's disease (AD). Solid lines show the AUCs for Fine-Gray and cause-specific models, and dashed lines show 95% bootstrap confidence intervals. Both models include apolipoprotein (*APOE*) ε4-residualized leukocyte telomere length (rLTL) interactions, adjusted for high cholesterol, pulse pressure, plasma glucose, erythrocyte sedimentation rate, lymphocyte proportion, age, age squared, and significant time-interactions. Cross-validation was based on 100 bootstrap samples. An AUC above 0.8 indicates a model with good discriminatory accuracy [1].

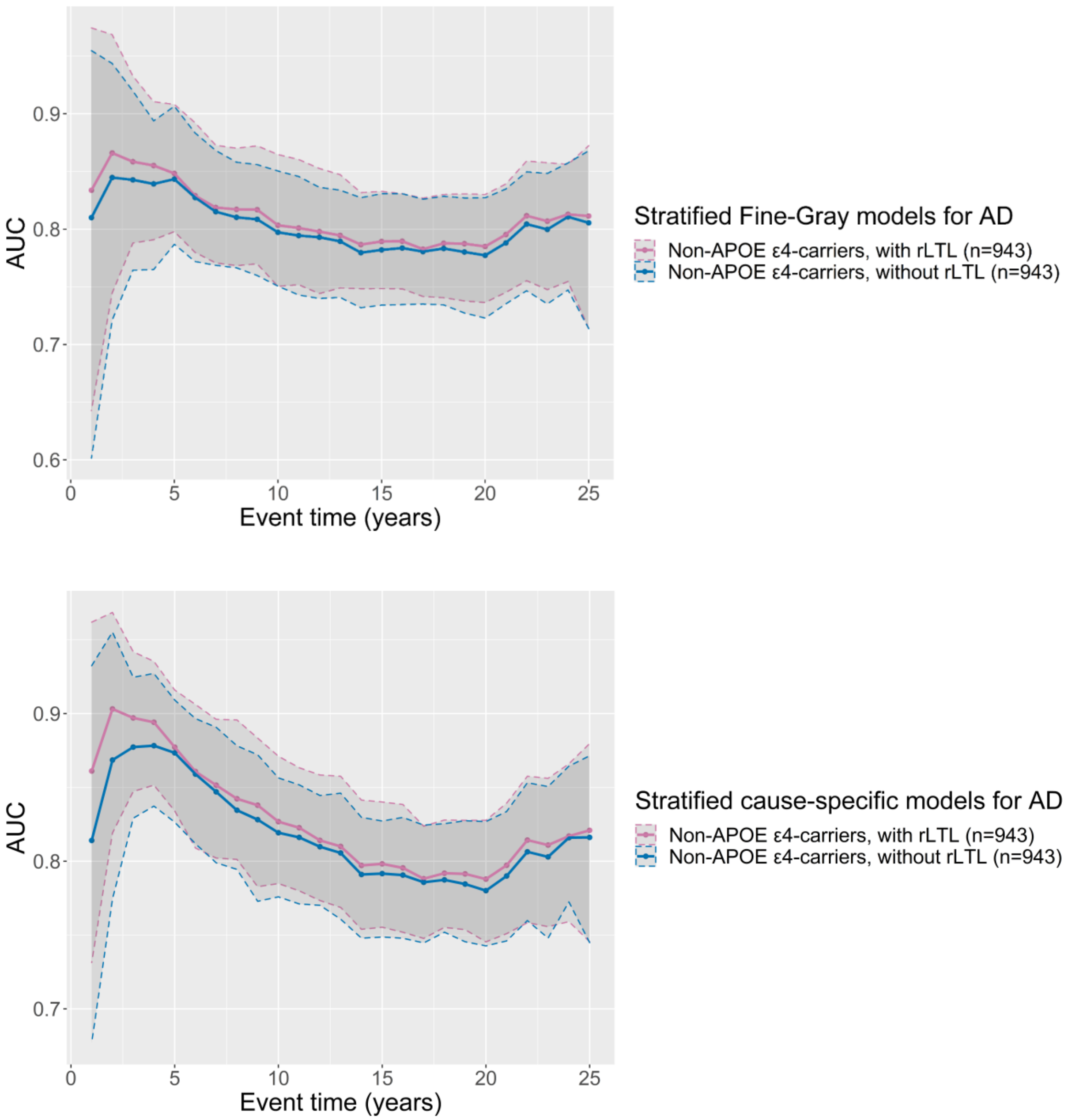

Supplementary Fig 4. Area under the receiver operating characteristic (ROC) curve (AUC) over the study time-course, showing the increase in prediction ability for Alzheimer's disease (AD) by residualized leukocyte telomere length (rLTL) in non-apolipoprotein (*APOE*) ε4-carriers (*n* = 943). Solid lines show the AUCs for the models, and dashed lines show 95% bootstrap confidence intervals. Fine-Gray and cause-specific models for the stratified non-*APOE* ε4-carriers subset are compared with and without rLTL, and adjusted for high cholesterol, pulse pressure, plasma glucose, erythrocyte sedimentation rate, lymphocyte proportion, age, age squared, and significant time-

interactions. Cross-validation was based on 100 bootstrap samples. An AUC above 0.8 indicates a model with good discriminatory accuracy [1].

Models' equations

The models considered in this study are two time-to-event regression models, based on the following proportional hazards

$$h_k(t) = h_0(t)e^{\beta_k X_k + \alpha_k^T(t) Z_k},$$

where *t* is time from baseline in years, $h_0(t)$ is the baseline hazard function, β_k are the coefficients for the accompanied covariates X_k, $\alpha_k^T(t)$ are the time-varying coefficients for the accompanied covariates Z_k, and *k* denotes the transition state (e.g. the transition from healthy to dementia). Here, we are primarily interested in the transition from a healthy to a dementia state while accounting for the competing events. Although the time-to-event models can be specified in a similar way, the *Subdistribution* and the *Cause-specific* models are different in how the hazard functions are estimated and the considered risk set at each time point [2].

<u>Subdistribution (Fine-Gray) hazard function</u>

The *Subdistribution (Fine-Gray) hazard function* denotes the instantaneous risk of occurrence from the *kth* type of event in subjects who have not yet experienced an event of type *k* [2, 3], and is defined as

$$h_k^{fg}(t) = \frac{Prob(t<T\leq t + \Delta t, D=k \mid T>t \cup (T<t \cap K\neq k))}{\Delta t} = \frac{f_k(t)}{1-F_k(t)},$$

where T is the time until event, D is event type, K is a collection of previously experienced event types, f_k and F_k denote the incidence function and cumulative incidence function for the *kth* type of event, respectively.

Cause-specific hazard function

The *Cause-specific hazard function* denotes the instantaneous rate of occurrence of the *kth* event in event-free subjects [2], and is defined as

$$h_k^{cs}(t) = \frac{Prob(t \leq T < t + \Delta t, D = k \mid T \geq t)}{\Delta t} = \frac{f_k(t)}{1 - F(t)} .$$

Cumulative incidence function (CIF) in the presence of competing risks

For the Fine-Gray model*,* the cumulative incidence function for event type *k* is

$$F_k(t) = 1 - e^{\int_0^t h_k^{fg}(s)ds}$$

[3, 4].

References

1. Zhang ZH, Cortese G, Combescure C, Marshall R, Lee M, Lim HJ, et al. Overview of model validation for survival regression model with competing risks using melanoma study data. Ann Transl Med. 2018. https://doi.org/10.21037/atm.2018.07.38.

2. Austin PC, Lee DS, Fine JP. Introduction to the analysis of survival data in the presence of competing risks. Circulation. 2016. https://doi.org/10.1161/circulationaha.115.017719.

3. Fine JP, Gray RJ. A proportional hazards model for the subdistribution of a competing risk. J Am Stat Assoc. 1999; 94(446):496.

4. Zhang ZH. Survival analysis in the presence of competing risks. Ann Transl Med. 2017. https://doi.org/10.21037/atm.2016.08.62.
